# Pennsylvania State Veterinary Medical Association

**Published:** 1893-10

**Authors:** 


*Pennsylvania State Veterinary Medical Association.*—The second day's session was convened at 10:15 A. M. President Hoskins in the chair.

On roll call—Drs. Allen, Bachman, Bartholomew, Benner, Ferley, Formad, Gladfelter, Glass, Goentner, Harger, Jno. R. Hart, Helmer, F. F. Hoffman, Hoskins, Keelor, Keil, Kooker, Lusson, Magee, J. C. Michener, Minster, Nunan, Pearson, Geo. B. Rayner, James B. Rayner, Thomas B. Rayner, John B. Rayner, Reinhart, Ridge, Ross, Sallade, Schrieber, Timberman Tintsman, Weaver, Weber, Webster, Cullen, Conard, Larzalere, Entrekin, Lintz, Houldsworth and Miller.

As visitors—Dr. William Dougherty, Baltimore, Md., of the Maryland State Veterinary Medical Association; Dr. H. P. Eves, Wilmington, Delaware, delegate from Keystone Veterinary Medical Association; Dr. E. O. Shakespeare, late of the City Board of Health; Dr. James McAnulty, Dr. Leo Breisacher, Dr. M. W. Drake, Dr. N. M. Drake, and a number of students from V. D. University of Pennsylvania.

The President appointed the following delegates to the various Veterinary Associations in adjoining States: To the New Jersey State Veterinary Medical Association—L O. Lusson, R. G. Webster and Walter R. Hart. To the Veterinary Medical Association of New Jersey—Drs. James B. Rayner, W. S. Kooker and P. M. Minster. To the New York State Veterinary Medical Association—Drs. J. H. Timberman, C. C. McLean and J. B. Irons. To the Maryland State Veterinary Medical Association—Drs. S. E. Weber, W. H. Ridge and Leonard Pearson To the United States Veterinary Medical Association—Drs. J. C. Foelker, Geo. B Rayner and John R. Hart.

Under discussion of reports, Dr. E. O. Shakespeare was called upon by the Chair to consider that part of the Legislative Committee's report pertaining to milk legislation. He addressed the Association at some length, answering every argument that had been advanced against the proposed law, touching upon the standard of quality as to solids; as to reduction by water and separator slop; the penalties of violation; the question of milk supply and the unfairness of many analyses; the wisdom of allowing the sale of skim milk and many other well-taken points, handling them all in an earnest, careful and familiar manner, gratifying and encouraging all who were present. After extending him a cordial vote of thanks for his interest and attendance, the milk bill and proposed amendments to other laws on the food question were unanimously indorsed.

Under the discussion of the report of Committee on Intelligence and Education, it was on motion approved that a committee of three be appointed by the Chair to draft suitable resolutions condemning the new two years' school at Washington, and extending congratulations to the American Veterinary College for its adoption of an obligatory three years' course. The Chair appointed the following committee—Chairman, Leonard Pearson, S. E. Weber and J. C. Michener. Said committee subsequently reported the following draft of resolutions:

Resolutions adopted at the annual meeting of the Pennsylvania State Veterinary Medical Association, Philadelphia, March 8th, 1893:

WHEREAS, Some of the officers of the Bureau of Animal Industry have established a veterinary school in Washington, which is poorly equipped and requires but two years' attendance upon instruction; and

WHEREAS, It is the opinion of this Association that all colleges awarding the degree of veterinary surgeon should give a course extending over at least three years; be it

*Resolved*, That we greatly regret the action of these officers and feel that they are doing the veterinary profession in this country an injury and are retarding the progress of veterinary education; be it further

*Resolved*, That a copy of these resolutions be sent to the honorable Secretary of Agriculture and to the Chief of the Bureau of Animal Industry.

SECOND RESOLUTION.

WHEREAS, Certain veterinary colleges have, to their own financial injury, adhered to three-year courses of instruction and others have discarded the old two-year course and now require of their students three years' attendance; and

WHEREAS, It is only through self-sacrificement of this sort that the cause of veterinary education can be advanced; be it

*Resolved*, That we commend these colleges having a curriculum covering three years, and also the American Veterinary College which has recently lengthened its course to this standard.

(Signed) LEONARD PEARSON,<br>S. E. WEBER,<br>J. C. MICHENER, } Committee.

At this point letters of regret were announced from Drs. C. P. Lyman, Isaiah Michener, D. C. Stanton, A. Liautard, C. R. Good, J. C. Foelker, Thos. J. Edge, and many others.

Under discussion of the Secretary's report, he was directed to remove from the rolls Drs. C. J. Blank, of Buffalo, on the ground of non-residence, and J. C. Thompson. To procure two hundred copies of Constitution and By-Laws.

It was further recommended that the Board of Trustees be requested to con vene an hour earlier, so that the bulk of business may be disposed of before the regular session convenes.

The Secretary was requested to comply with the desire of Dr. A. H. Derney, a non-resident, who wished to recall his name.

The discussion of the Treasurer's report, it was decided to have the following names stricken from the roll:

Drs. C. A. Miller, S. K. Hoffman and A. F. Schrieber. Subsequently, on payment of initiation fee and dues, Dr. Schrieber was reinstated. All bills of Secretary and others were referred to an Auditing Committee, consisting of Thos. B. Rayner, W. S Kooker and S. J. J. Harger, after which adjournment for lunch.

The first paper presented to the Association was by Prof R. S. Huidekoper, of New York City. entitled "Agricultural Shows, Judges and the Veterinarian," which was listened to with the most intense interest and edification, and proved to be a paper of exceptional merit, strength and importance, and suggested a new field of labor for the veterinarian to fill that gives great promise of strengthening them as a profession in the eyes of our people throughout the entire country. So carefully and thoroughly was the paper prepared, and so wholly new its character, that its completeness forbid its discussion, and the Association could only most generously thank the author for his kindness in preparing it for the meeting, with the desire that our Association should give it the publicity it should command (*a*).

The second paper, read by Dr. J. Curtis Michener, on "Open Joints," proved a short, terse article on this topic, specially considering his plan of treatment. The paper was afterwards discussed and many questions were asked in regard to the result of the plan of treatment outlined (*b*).

This paper was followed by one on "Fungus Hæmatodes in Cattle and Horses," by Dr. James A. Waugh (*c*). He being absent from the meeting, the paper was read by the Secretary. This was followed by a paper on "Acute Toxic Anemia," with the reports of some animals dying from this cause, by Dr. Jacob Helmar, of Scranton (*d*). This paper proved to be of exceptional interest, and was listened to with a great deal of pleasure by all present. His investigation of his case had been of the most thorough character, and the entire history of the development, progress, results and probable origin of the disease was prepared to a state of completeness that won for the author the admiration and approval of all who had the pleasure of listening to his paper.

This paper was followed by one by Dr. Leonard Pearson, continuing the subject of "Tuberculosis" and the results obtained by the use of tuberculin as a diagnostic agent. Some other important and valuable statistics were thus added to what he had placed on record some six months before, and all pointing to the increased value of tuberculin for the detection of tuberculosis in the bovine species. He exhibited, in conjunction with his paper, several specimens that had been obtained from cattle where the symptoms had been peculiarly interesting and somewhat obscure. His paper elicited much interest and brought forth many inquiries that exhibited the general interest shown by the veterinarians in the consideration of this important subject.

As a contribution in connection with the paper of Dr. Pearson, Dr. W. B. E. Miller of the Bureau of Animal Industry, chief examiner of cattle for foreign shipment at the port of Philadelphia, and who with his corps of assistants were continually seeking for evidence of the existence of tuberculosis among the animals selected for consumption as food in Philadelphia, reported the results of these investigations for the past year, which shed much light upon the healthfulness of the beef supply at this centre.

---

(*a*). *Vide, fol.* 129. (*c*). *Vide, fol.* 156.

(*b*). *Vide, fol.* 160, (*d*). *Vide, fol.* 150.

This closing the list of papers, the remaining short time of the meeting was thrown open for reports of cases, one of which, reported by Dr. W. Horace Hoskins, was a case of enormous calculi of both kidneys of a Great Dane bitch. The entire structure of one kidney had become entirely broken down and the walls of the organ, greatly distended, had simply become the surrounding envelope of an enormous cyst, containing the calculous deposits; the other had still, to a certain extent, maintained its shape and normal size and containing within an extremely large deposit. The animal had died from uræmic poisoning, indicated principally by great depression of all the vital forces, profound loss of appetite, great lethargy, but never evincing any evidence of acute pain or suffering.

The place for holding the semi annual meeting was then brought up for consideration and resulted in the selection of Scranton. A hearty vote of thanks was then tendered to all those who had contributed to the interest and pleasure of the meeting through the papers and reports which had been submitted. The seating of the newly elected officers then took place, and a vote of thanks to the officers of last year was then accorded after which the meeting adjourned.

Dr. Robert Gladfelter, *Recording Secretary.*

W. Horace Hoskins, *President.*

---

### COMMITTEE REPORTS.*

*Committee on Legislation.*

Mr. President and gentlemen:—The Committee on Legislation beg leave to report that since our semi-annual meeting we have had a suit brought before the courts of Washington County, by one of our committee, against Dr. Rogers, of Washington, Pa., for false registration, having registered as a graduate of a college which never had an existence as a veterinary college, nor ever issued a diploma conferring the veterinary degree. The costs in the suit were equally divided between the prosecutor and defendant on the ground of limitation, the time between the registering and the suit being over two years. The committee suggests the inadvisability of bringing any more suits unless they are plain and very recent violations of the law.

Lawing is very expensive, and money thus expended might be used in the publication of literary matters that would be of more importance and benefit to the profession. The committee expended sixty-five dollars in the Washington County courts. We would recommend the earnest support of the following bills and amendments now before the Legislature, or in contemplation, and hope that every member will take a personal interest in the work, not only in his own county or district, but will work with veterinarians in other counties, or with the representatives direct, and keep our Corresponding Secretary posted in regard to what you are doing and the result of your efforts.

W. S. Kooker,
Thos. B. Rayner.

---

*Report of Committee on Intelligence and Education, by Dr. W. E. Weber, Chairman.*

Since our last meeting there has been evident increase of feeling on the part of members of our profession, touching upon improvement in the standard of education in that department of medical science in which we are most directly interested. The concensus is for a class of men better equipped than heretofore for the duties de-

*Read before the annual meeting of the Pennsylvania State Veterinary Medical Association, March 7th, 1893.

volving upon them as physicians for the faithful creatures in our care that are unable to make known to us in words their needs as sufferers, as well as other branches of the science. This better equipment, of course, means better rudimentary education, supplemented and strengthened by scientific training in a course which, according to sound thought, should not be ended until the student is thoroughly qualified to enter upon his career as a veterinarian.

It is claimed by some—and with justice—that no man should be sent forth to practice as a veterinarian unless he has proved not only that he has mastered the details of his profession, but that he has a pride in it, and is actuated by a desire and a determination to advance it. In other words, that the veterinarian, like any other true scientist, should be an enthusiast.

The student of veterinary science should be an enthusiast, as without doubt he should be a lover of animals and in full sympathy with them. It is not too much to say that the most successful veterinarian, all other things being equal, is he who has studied the habits of animals, has learned their peculiarities, has become versed in their moods, and at all times feel kindly towards them. We all know the magnetic influence of the physician upon his human patient; we know that when the sufferer feels that his doctor is in full sympathy with him, the patient has a much better chance to recover from his ailment than if between him and the medicine man there were no bond stronger than a porous plaster and a greenback. You may call the good effect the effect of imagination, if you will, or give it any name you please, but the effect is there all the same. It is just so in the case of brute patients, and no observing veterinarian will deny it.

There are now some twenty regularly chartered veterinary schools and colleges in the United States and Canada, but there are a number of institutions ostensibly veterinary schools, which, in great part, are not legitimately conducted. I do not here mean to say that they are not conducted according to the laws enacted for the government of such institutions. As a general thing, they are founded and managed not for the benefit of veterinary students, but mainly in the interest of individuals whose prime object is self-aggrandizement, especially in the direction of money-making. A large majority of these offer a complete course of instruction after an attendance of two years. Now, notwithstanding the fact that until a comparatively recent period, nearly all the schools required but a two years' course to complete an education in veterinary science, all our best practitioners knew that it was impossible to gain a thorough knowledge of it in so short a term. So, as the influence of the profession increased, the wisdom of its leaders began to have its effect. Their protest against ramshackle methods was harkened to; when they gave cogent reasons why the acquirement of instruction in veterinary principles required more time than was devoted to it, men weighed those reasons and did not find them wanting, and at last the leading schools extended their term to three years.

The good result of this praiseworthy action is already apparent. The class of students sent forth from the schools is of a higher grade than ever before, and their equipment is immeasurably better than it was when the curriculum was run through from title page to colophon in little more time than should have been devoted to the mere alphabet of the science.

That there has been no falling off in the number of students graduating with full honors from these colleges is shown in the fact that in all of these colleges whose term has been extended, the list of matriculants has regularly increased; although the expense to each student is one-third greater than it was when the diploma could be secured after only two years of work. A few remarks from a distinguished colleague (Dr. Austin Peters), who was Chairman of the Committee on Intelligence and Education of the United States Veterinary Medical Association in 1890, may here be quoted as being directly upon this subject :

"In 1863, when this association was first organized, it consisted chiefly of men who were non-graduates ; there were very few members of the association who were graduates of veterinary schools, because there were no veterinary schools in the country from which they could graduate, and the few having diplomas obtained them by pursuing a course of studies abroad.

"Yet these non-graduates were, as a whole, honorable, conscientious men, good practitioners of their profession ; they were fairly well read, and enjoyed the respect and confidence of the communities in which they lived. But a change has gradually taken place in the *personnel* of our association. Many of the old non-graduates have passed away and but a few remain ; and the numbers who have joined us of late years have all been graduates of veterinary colleges. To-day it would be an impossibility for a non-graduate to become a member of our body."

Had Dr. Peters been writing this in 1893 he could have had the pleasure of stating the wise action of that body in requesting still more from its applicants for membership, in that it now even requires a man to be a graduate of a school having a curriculum extending over a course of studies of not less than three years. A most progressive step in the advancement of our profession.

He further says: "This change, however, was brought about in a great measure by these non-graduates themselves. . . . . These men asked for something better and they got it. The question now propounds itself to us, shall we be satisfied with our veterinary schools as they are, or shall we not, in this great age of progress, ask for something better—a longer, more thorough course of study, with a higher educational qualification for matriculation, and a higher standard of graduation than at present prevails?" Your committee would recommend that the members of this association recognize the action taken by the United States Veterinary Medical Association in adopting the amendment which was so unanimously carried at their last meeting in Boston, by doing something towards passing a like amendment, which is as follows :

"ARTICLE I.—Any applicant for membership shall submit his name upon one of the association's application blanks, duly vouched for by one or more members of the association, or by the resident State Secretary of his respective State. He shall be a graduate of a regularly organized and recognized veterinary school, which shall have a curriculum of at least three years, of at least six months each, specially devoted to the study of veterinary science, and whose corps of instructors shall contain at least four veterinarians. If of a medical school, a similar curriculum as to time shall prevail."

This amendment does not apply to matriculants of the year 1892, nor shall it apply to applicants who were college matriculants prior to its passage.

There is no act on the part of veterinary medical associations that can do more towards stimulating the cause of improvement in veterinary educational matters in America, than the passage of such requirements for admission. It is with just pride that we hail the announcement of the American Veterinary College of New York, that on and after 1893 her course will be an obligatory one of three years. On the other hand, it is with extreme regret and disappointment that our attention is drawn to the fact, that there has recently been established at the Capital of the Nation a evterinary college whose course of study is but two years. When we consider that this college is under the protecting wing of the Government Bureau of Agriculture, that its faculty is composed of eminent men who have always advocated "a longer, more thorough course of study, with a higher educational qualification for matriculation, and a higher standard of graduation than at present prevails," what will we have to say about the necessity of providing safeguards against the admission of the two year graduates as members of this association? A two year course does not provide for a graduate, higher educational qualifications. I agree that "a good preparatory course for the future veterinarian is to attend one of our agricultural colleges;" but the preparatory course does not complete the student's equipment, nor does a two years' course in any veterinary college in the land.

The question. "Shall we be satisfied with our veterinary schools as they are?" is one that should be as carefully considered to-day as though it had reference to a date twenty years in the past. We should not be satisfied with them so long as it is possible to improve them; and that there is plenty of room for improvement is a fact as patent as that the improvement is needed.

It is time now for a veterinarian entering upon the duties of his calling, to come thoroughly equipped for his work; but where is the place to which he can turn for such thorough equipment? At one school he can be supplied with the rudiments of an education; at another he can learn to treat, with some degree of skill, diseases of the horse; in this college he can become a good surgeon; in that he can be fitted out with all the adjuncts of the science; while there are other institutions in which he can gain a smattering of everything pertaining to his profession, simply by listening to a series of lectures, delivered in some cases by "professors" whose knowledge of veterinary science is confined to the text-books from which they quote with refreshing liberality. Such schools will exist in spite of all the best efforts of the profession to abolish them; but it is in our power to mitigate the evil of their existence; and that power lies in our ability to *taboo* them—to ignore them—by refusing absolutely to recognize their alleged graduates as legitimate members of the veterinary profession, and consequently debarring them from the privilege of identifying themselves with this or any other standard association devoted to the interests of veterinary science. If the State and local associations follow the precedent set apart by the United States Association, some good towards the springing up of institutions of low standard must be brought about. We must become stronger in our recognition towards the schools whose intentions are for the good of the profession. We must bring about influence to show the student of to-day where he can go to become thoroughly equipped.

The rapid multiplication of veterinary schools still goes on, and those that have been recently organized are, one at Detroit, Michigan; Cincinnati, Ohio; Des Moines, Iowa; Kansas City, Missouri, and the latest one at Washington, D. C.

In reference to the announcement of the organization of this last-named school, we can only say that it is one of the sorest disappointments that could confront the more advanced thought of the profession at this time, to say the least of it. And we hope that the instigators of this new institution will reconsider their action and by the next announcement we may see the Capital of the Nation adorned by one of the standard veterinary schools of the world. There is certainly no other place in this country that can offer better facilities for such a purpose than Washington. It is incumbent upon us to-day, as an association, to denounce the establishment of this two-year school by proper resolutions of condemnation. Much has been said and with just opposition, by the profession since its announcement.

Resolutions showing the disapprobation of the profession have been sent to the proper United States authorities. as well as the officials of the Bureau of Animal Industry, by the Philadelphia Veterinary Medical Association, the Massachusetts Veterinary Medical Association. the Keystone Veterinary Medical Association of Philadelphia, and the New York State Veterinary Medical Association It is the duty of the profession in this country to be a unit in trying to bring about the desired result. Leaving the subjection for the consideration of this body to-day, I will return to another subject of improvement in education, namely, the unscrupulous practitioner, by whom we have been long enough impeded. While we acknowledge that there are good and conscientious veterinary practitioners who have assumed the duties of professionals without proper educational equipment, we must regard them as rare exceptions to the general rule and continue to look upon the majority of the haphazard "horse doctors" in the light of charlatans, who should be forced by lack of patronage to throw up their trade and take to something for which they are better fitted by nature, education and experience. Time was, even within the memory of men not yet old, when the human, as well as veterinary, powwow doc. tor in many Pennsylvania communities; when by blowing his fetid breath upon a wound, and reciting a gibberish abracadabra to the ignorant and awestruck patient, he could make him believe that his ailment was cured, and that by the going down of the next day's sun nature would give proof of the wonder-worker's power by closing up the cruel gap and covering it with an epidermi not only as healthy as an infant's, but as tough as the hide of a bull of Bashan. There are still among us relics of the past who have an abiding faith in the efficacy of the powwow doctor's magic treatment, and would pit one blast of the foul exhalations against all the saving drugs in the pharmacopœa; or one syllable of his meaning less jargon against the *dictum* of the wisest and most humane physician that ever administered paragoric or lanced a gum. But, brethren of the pill and plaster, *where is the powwow man to-day?* Gone. like the unsubstantial vision that has faded, he has "left no wrack behind," except the rack that pinches the conscience of the man who remembers that he once pinned his faith to powwow; and that he believed there was almost divine omniscience in the powwow man's words uttered through a rum-flavored breath. Yes, he is a thing of the past. The bright light of education has fallen upon the shade of ignorance and superstition, and we hope the sun will never again shine upon the powwow-man or any other retrograde barrier, so that his shadow shall not darken the pathway of improvement in any of the branches of medical science.

I am glad to believe that before long the veterinary quack and charlatan will be relegated to obscurity by the same influence.

QUACK ADVERTISING IN AGRICULTURAL JOURNALS.

The practice of our so-called agricultural journals, in giving great encouragement to the veterinary quack, is to be denounced; but what shall be said of periodicals like the *Spirit of the Times*, the *Turf, Field and Farm* and the *Horseman*, ostensibly devoted to the improvement and well-being of the horse, which sell columns of space every week to quacks and charlatans for the puffing of their nostrums? And right here let me enter my protest against the weekly publication, in journals such as I have named, of a department under the head of "Veterinary." This department is supposed to be conducted by a reputable veterinarian; but what must be the nature of the information he imparts to correspondents he has never met concerning the ailments of animals he has never seen? Is not this a species of cheap quackery? Is it not dishonest? It is certainly not of benefit to the subscribers of these journals, how much soever it may seem to be a gratuitous service.

These journals—and there are many of them in the United States—are not published in the interest of veterinary science, it is true; but we have a perfect right to criticise their conduct in this regard, at least. Their advice on subjects pertaining to the diseases of animals is not the kind that should be given, for the reason that it is based upon an indistinct, faulty or absolutely wrong impression on the part of the horse's guardian, who, in a vast majority of cases, must be unprepared to diagnose a disease, and is not able to make plain (in writing) what he has discovered as to the animal's condition. What sort of advice, then, does the literary veterinarian give in answer to the rambling, disconnected and almost unintelligible account of the animal's trouble as set forth in his inexperienced correspondent's letter? What cruel blunders are made by stock-owners of limited intelligence who attempt to carry out the instructions of the pen-and ink practitioner who issues his edict five hundred miles from the patient's stall. Put together the ignorance of the man who asks the advice and the boldness and self-conceit of the man who gives it. Let the two come to a result. Who can estimate the mischief? Take this one case, for example:

A farmer in the northern part of New Jersey had a valuable horse which, about six months ago, went wrong. In his distress the farmer, instead of summoning a competent veterinary surgeon to examine and treat his horse, wrote thus to the desk veterinarian of a well-known New York sporting paper:

"I have a valuable horse that is rough in his coat, goes lame in one hind leg once in a while, and has a discharge from the nose and sometimes also from the eyes. He has a swelling under the jaw, and the sheath is also swollen. He does not eat well, has a cough and gets weak after working a little while. What do you advise me to do? The pen-and-ink doctor, instead of saying, "Consult a good veterinarian," gave this reply: "The case you describe is in all probability one of glanders. I should advise you to give your horse at once a purgative—eight drachms of aloes in a ball, and if he gets much weaker he has glanders beyond a doubt." The foolish farmer, who had received this advice at the expense of his weekly agricultural and sporting paper, gave his horse the prescribed dose, and the animal, which was suffering from influenza and needed all his strength, was purged to death in two days.

Had the penny-wise and pound-foolish farmer secured the services of the nearest veterinary surgeon, instead of depending upon the opinion and advice of a quill-driving snap-chance, he might have saved his valuable horse at the cost of a few dollars, and at the same time learned a valuable lesson in veterinary science.

All that has thus far been said comes directly under that part of the head of education, whose province is improvement.

Now, the *status* of our profession can be elevated and improved by means of prompt and earnest action in the direction already pointed out. Yet something more is needed.

We recognize with something of shame, mingled with our indignation, that there are no sufficient laws in our State protecting our profession. Such laws have been passed, but found inadequate to bring the desired effect; but amendments are in progress, and we hope, if passed, will be duly enforced, and thus save the public as well as the profession, who are at the mercy of empirics and veterinary tramps, who not only swindle the public and the honest members of our profession, but by their ignorance also lower the profession in the eyes of the untutored world. The peripatetic "horse doctor," who tramps from stable to stable uttering platitudes in a jargon more startling, but less intelligible than dog Latin, and treats every ailment with the nauseous compound poured from the one bottle belonging to his medicine chest, cannot, of course, impose upon an intelligent man. His dupes are of the class who need protection from these confidence operators. They are the unfortunates who do not read the papers and are too ignorant to profit by the unhappy experience of other ignoramuses.

There are two means by which these frauds may be prevented. One is to eradicate the evil by driving out the fraud; which can be done by bringing into play the strong arm of the law. But if we could only apply the second means and educate the army of dupes up to a knowledge that these peripatetic practitioners are ignorant empirics and unable to do them justice, the aid of the law would no longer be required.

There are laws in various States to repress this class of practitioners, but are these laws enforced? True, it is not fully as easy for the veterinary quack to practice in 1893 in the *protected* States as it was half a century ago, when there was hardly a veterinary school in the United States, and when the Statute Books were bare of any reference to bogus practitioners. These laws are practically a dead letter—as lacking in life as the plaster model in a dressmaker's window. It behooves the legitimate veterinarian to discuss the question on all its bearings, and let such laws be framed by competent hands and submitted to the Legislature with a plea for their enactment.

Let every honest veterinarian in the interest of his profession, for the protection of the stock-owner and for the safety and better treatment of the dumb victims of the quack's inhumanity and ignorance, *insist* upon the rigid enforcement of those laws.

Let him call attention of the local authorities to each infraction, and demand the arrest of the malefactor and aid in having punishment meted out to him.

The veterinarians are not sufficiently numerous to form a political party, to be sure, but there are 1,000 in the State of Pennsylvania, and they are good members of society; and besides a vote, each one has more or less influence as a citizen, and an educated man, in shaping the political sentiment of his neighbors. This is a fact that members of the Legislature may some time deem of sufficient importance to make a note of.

The importance of co-operation on the part of veterinarians in effecting reforms cannot be overestimated. It should be borne in mind that every reform as pertaining to our profession, its *status*, its advancement, its protection, bears directly upon the welfare of the general public. You cannot act for or against a profession without helping to retard or expedite the progress of the whole community in one direction or another. If you weaken the barriers that keep out the quacks you leave the public at their mercy. If you combine in a movement to make scientific investigations into the prevalence of a disease, you do for the public a service worth millions of dollars, besides providing a safeguard against future plagues which, unchecked, might carry off the greater portion of the live stock in the country, and leave its inhabitants subject to transmitted diseases.

When cholera, smallpox, or any other contagious disease threatens us, we are prepared with the means whereby the approach of the plague may be rendered impossible. To what is this due? To the concerted action of wise, thoughtful, public-spirited physicians, who, without any reference to political bias or personal aggrandizement, move together to bring the law into operation that it may work hand-in-hand with science for the general good.

Every veterinarian should be an active member of some branch of the great Society for the Prevention of Cruelty to Animals, and should use his influence to establish other branches wherever he may see the need of them. This I consider a part of his education, as well as a very necessary part of the curriculum of every member of society, whether a professional or not. In maintaining such societies the veterinarian has a facility possessed by no one else, for he not only understands animals and their needs, but can instruct others as to the best methods of relieving the suffering brute, and in various ways rendering an overworked or wounded animal comfortable. His knowledge enables him to make his sympathy for dumb animals doubly felt and appreciated, and in every community he becomes a benefactor, not only to the brute but to his fellow-man.

Now that all the eyes of America are turned to Chicago to watch the progress in the preparation for the great Exposition, it behooves us as members of a profession acquiring importance with its growth, and holding an honorable place where it was once too generally the object of contempt, to consider what part we are to take in the coming celebration. It has been decided to hold the first International Congress of Veterinary Science there, and no one will deny that it is the duty of every member of this, the Leading State Association of the United States, to be present. The occasion may be, and should be, made the most eventful in the history of our profession. There will be, then, the largest gatherings ever known here or in Europe; the best minds of the profession will be there to aid and encourage us by the interchange of thought; experiences will be brought forth that will increase our knowledge and strengthen our loyalty to a noble calling, and by our presence we will be factors, giving dignity to the effort we make to elevate the standing of our profession

The 1,800 or more veterinarians in the United States should be there in force, not as mercenary onlookers, but as earnest workers in a field in which the standard of professional worth should be as high as that of any science known to man.

At this International Congress we shall be afforded an opportunity to make an advance in the improvement of medical science that should have been made long years ago. I mean an amalgamation of what we may call human and veterinary medicine, as the practice of the science of medicine for man and the lower animals is so closely related by nature that it is difficult to understand one without a knowledge of the other. The amalgamation may be brought about by arranging the subject matter of the various discourses so as to make them interesting to the medical fraternity, by showing them how intimate are the relations existing between our practice in the stable and the barn and theirs in the bedchamber and the hospital. There is no doubt that the two professions—if two there may be said to be—have been kept assunder without good reason.

To emphasize the suggestion just made, let me quote a few words addressed by Prof. Woodhead, a celebrated physician, at a recent meeting of the National Veterinary Association, in Edinburgh, Scotland. Prof. Woodhead had had some experience as a veterinarian, and a vote of thanks had been tendered him for a paper he had read before them. This is part of what he said :

"I am free to say, from my own point of view, that it has been a great pleasure to me, and a great profit, likewise, to work professionally with you. I have derived a great deal of knowledge from my association with Prof. McFayden, V.S., knowledge that I could not have gained had I not engaged in his kind of work. I have learned to know how close the two professions are, how closely connected are medical science and veterinary science, and I am sure that if you would only meet more frequently and interchange ideas more freely, it would be of untold advantage to both branches of the healing profession."

